# The Fast Spiking Subpopulation of Striatal Neurons Coding for Temporal Cognition of Movements

**DOI:** 10.3389/fncel.2017.00406

**Published:** 2017-12-15

**Authors:** Bo Shen, Zuo-Ren Wang, Xiao-Ping Wang

**Affiliations:** ^1^Department of Neurology, Shanghai Tong-Ren Hospital, School of Medicine, Shanghai Jiao Tong University, Shanghai, China; ^2^Institute of Neuroscience, State Key Laboratory of Neuroscience, Shanghai Institutes for Biological Sciences, Chinese Academy of Sciences, Shanghai, China

**Keywords:** timing dysfunction, subpopulation of striatal neurons, fast-spiking neurons, procedural learning, movement disorders, Parkinson’s disease, autism

## Abstract

**Background:** Timing dysfunctions occur in a number of neurological and psychiatric disorders such as Parkinson’s disease, obsessive-compulsive disorder, autism and attention-deficit-hyperactivity disorder. Several lines of evidence show that disrupted timing processing is involved in specific fronto-striatal abnormalities. The striatum encodes reinforcement learning and procedural motion, and consequently is required to represent temporal information precisely, which then guides actions in proper sequence. Previous studies highlighted the temporal scaling property of timing-relevant striatal neurons; however, it is still unknown how this is accomplished over short temporal latencies, such as the sub-seconds to seconds range.

**Methods:** We designed a task with a series of timing behaviors that required rats to reproduce a fixed duration with robust action. Using chronic multichannel electrode arrays, we recorded neural activity from dorso-medial striatum in 4 rats performing the task and identified modulation response of each neuron to different events. Cell type classification was performed according to a multi-criteria clustering analysis.

**Results:** Dorso-medial striatal neurons (*n* = 557) were recorded, of which 113 single units were considered as timing-relevant neurons, especially the fast-spiking subpopulation that had trial–to–trial ramping up or ramping down firing modulation during the time estimation period. Furthermore, these timing-relevant striatal neurons had to calibrate the spread of their firing pattern by rewarded experience to express the timing behavior accurately.

**Conclusion:** Our data suggests that the dynamic activities of timing-relevant units encode information about the current duration and recent outcomes, which is needed to predict and drive the following action. These results reveal the potential mechanism of time calibration in a short temporal resolution, which may help to explain the neural basis of motor coordination affected by certain physiological or pathological conditions.

## Introduction

Given the dynamic nature of the world, estimating duration and predicting its consequences is a fundamental skill for the daily activity of organisms. This timing ability is of critical importance to inter-temporal decision-making, sensory integration and motor coordination (Buonomano and Laje, 2011). It is thought that these distinctive functions are processed in distributed cortices, with extensive evidence suggesting that the primary sensory cortex (Namboodiri et al., 2015) and motor cortex (Li et al., 2016) convey time information in behavioral prediction, in addition to their functions in encoding sensory or motor features. Specifically, motor outputs require precisely programmed actions timed in sequence, from a muscle group to another or guided by external sensory cues, such as playing a musical piece, shooting a basketball, and waiting for the sound of a gun to start a race (Buhusi and Meck, 2005; Merchant et al., 2013). In some pathological conditions action sequences are not well orchestrated, such as stereotyped behavior in autism (D’Cruz et al., 2009) and impulsive behavior in attention-deficit-hyperactivity disorder (ADHD) (Valera et al., 2010; Nieuwboer and Giladi, 2013), due to disturbances in temporal cognition.

The striatum is involved in reinforcement learning and procedural motion. The striatum is the main input layer of the basal ganglia, which receives glutamatergic inputs from multiple cortices and dopaminergic inputs from substantia nigra compacta (SNc). These connections place striatal neurons in a unique position to integrate divergent contextual and kinematic information (Reig and Silberberg, 2014; Rueda-orozco and Robbe, 2015). Several lines of evidence implicate the striatum in encoding timing on seconds to minutes range. Impaired duration discrimination has been reported following dysfunction of the striatum, such as in Parkinson’s disease (Parker et al., 2013) and pharmacologically damaged laboratory animals (Meck, 2006). Human fMRI (Tanaka et al., 2004) and animal *in vivo* electrophysiology studies (Matell et al., 2003; Mello et al., 2015) have revealed that striatal neurons are activated when a subject is predicting a reward or action, and they sustain modulation of their firing rate until the time the action is executed.

In addition, the striatum can participate in neural processes at different time scales through different mechanisms (Buhusi and Meck, 2005; Meck et al., 2008; Jin et al., 2009; Harrington et al., 2011). For short periods, such as sub-seconds to a few seconds, timing is crucial for motor control, and it is considered an intrinsic property of neurons or achieved through neuronal circuits. However, an agreement has not been reached regarding the role of the striatum in timing on the sub-second to second scale. Recently, a rat study demonstrated that striatal ensembles are able to encode the judgment of sub-seconds (Medina et al., 2000). Despite this, it is still unknown how striatal activity can encode rats’ behaviors in the sub-second scale. In order to better understand how striatum encodes time and precisely coordinates distinct actions in a series of motions, we have designed the 1.5-s fixed-interval reproduction behavior task and have recorded the spike activity of dorso-medial striatal neurons from well-trained rats. Since the main types of striatal neurons can be distinguished from their respective waveforms and firing properties, we have identified putative fast spike neurons and observed that this type of striatal neurons presents homogeneous modulation during the timing process. Furthermore, we also found that the firing amplitude of striatal ensembles adapted to recent experiences of timing behaviors on the sub-second to second scale.

## Materials and Methods

### Subjects

Wild-type adult male Long-Evans rats weighing 300 to 400 g were used in this study, which was approved by Institute of Neuroscience, Chinese Academy of Sciences Animal Care and Use Committee (Certificate No: NA-110411). The procedures were performed strictly in accordance with the approved guidelines. The animals were raised with a 12-h light/dark cycle, and all training was scheduled at a specific time for each rat during the daytime.

### Fixed Interval Reproduction Task

**FIGURE 1:**
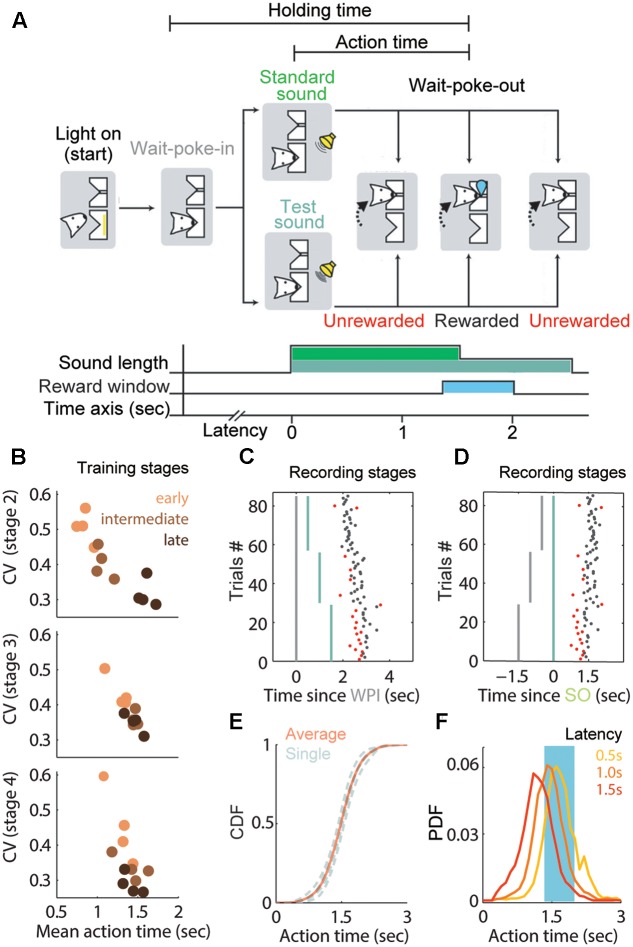
Adaptive waiting time and training performance in the 1.5 s fixed peak task. **(A)** Sequence and duration of task events in a trial. Rats were trained to reproduce a fixed interval from standard or test sound onset (SO) until the wait-port-out (WPO). This duration was defined as the action time (AT) and lasted 1.5 s following the SO (light green). Note that the sound length was prolonged to 2.5 s in the test trials (dark green). **(B)** AT performance of each rat at different stages. Each dot represents the coefficient of variation (CV) of the AT at early (orange), intermediate (brown) or late (black) phases in the stage. **(C,D)** Representative session showing the relation between WPI (**C**, gray line), SO (**D**, green line) and the corresponding WPO. Non-timed trials are shown in red. Most actions are timed from the SO in well-trained rats. **(E)** Cumulative distribution function (CDF) of ATs from the recording stage, gray curve for each rat and red curve for the average. **(F)** Probability distribution function (PDF) for the ATs separated by latency: 0.5 s (yellow), 1 s (orange) and 1.5 s (red). Same data as in **(E)**.

The behavioral chamber contained two nose-pokes, named “wait port” and “reward port.” The wait port was used for rats to poke in and estimate a fixed duration, whereas the reward port had a liquid tube delivering water on correct trials. For each trial, the wait port lighted up and the animal poked its snout into this port, an action that was defined as “wait-port-in” (WPI). The rat stayed in place for a semi-random non-repeat delay (0.5, 1.0, and 1.5 s). The trail continued with an acoustic cue, and then the rat had to estimate the 1.5-s duration and reproduce it by exiting the wait port, an action that was termed “Wait-port-out” (WPO). The time from “sound onset” (SO) to WPO was defined as the action time (AT), and the time from WPI to WPO was defined as holding time (HT) (**Figure 1A**). A trial was considered correct if the AT fell within 1.35–2.0 s. Rats were rewarded with water (100 μl) after poking into the reward port on correct trials. If rats failed to respond (omission trial) or exited prematurely (premature trial), white noise and a time-out (10 s) were used as punishment.

To train subjects to reproduce the specific duration precisely, we used an acoustic cue (2 KHz pure tone) of two different durations (Xu et al., 2014). The duration of the standard sound was the same as the target duration (1.5 s), and it was used as a reference instruction, whereas the test sound lasted 2.5 s. A session consisted of two alternating blocks; in the first block the 1.5-s instruction trials were repeated 20×, and in the second block the 2.5-s test trials were repeated 30×. Only data from the test trials were subjected to further analysis, because the rat’s exiting reflected the internal timing information more objectively (without guidance) by the offset of the acoustic cue.

### Behavioral Training Procedure

Water-restricted rats were trained sequentially for four stages before being taken into the final experiment (**Table 1**). At the first stage, the standard sound played and water delivery was triggered immediately when the rat entered into the wait port; meanwhile, the acoustic signal and reward could also be given automatically every 2 min. After subjects habituated themselves to poke the wait port and obtain water at the reward port (3.3 ± 1.2 sessions), they were required to hold the position once they poked into the wait port until the standard sound stopped in order to reinforce the correlation between the duration and a reward. If the subject exited from the wait port early, the sound continued until the set duration and no reward was delivered for that trial. According to each subject’s performance, the standard sound and duration could be increased gradually from 0.75 to 1.5 s across the first few days of this training stage (9.0 ± 0.8 sessions). In stage 3 training, a latency period was introduced before the acoustic signal. Most subjects could accomplish these initial stages within 3 weeks (21.5 ± 2.6). Subjects were then trained in the final training stage (same as the recording stage) in which they had to perform both 1.5-s instruction trials and 2.5-s test trials during the same session, until the AT in both kinds of trial reached a stable range around the 1.5 s duration (coefficient of variable, CV < 0.4 in at least two consecutive sessions, 25.0 ± 2.6 sessions).

**Table 1:** Training schedule of fixed interval reproduction task.

Training stage	Delay	Cue duration	Action time in rewarded trial	Duration of a session	Criterion to move to next stage
1	None	1.5 s	None	15 min	Correct trials > 50
2	None	1.5 s	>1.5 s	45 min OR > 200 trials	CV < 0.4 for two consecutive sessions
3	0.5∼1.5 s delay	1.5 s	1.35 s∼2.0 s	45 min OR > 200 trials	CV < 0.4 for two consecutive sessions
4	0.5∼1.5 s delay	1.5 s OR 2.5 s	1.35 s∼2.0 s	45 min OR > 200 trials	CV < 0.4 for two consecutive sessions

### Surgery

The rats were implanted electrodes once they learned the stage 4 training. Anesthesia was initiated with 4% vaporized isoflurane. Prior to injecting 2% pentobarbital sodium (0.4 ml/100 g), a small supplementary dose of atropine (5 ug/100 g) was given to inhibit glandular secretion during anesthesia induction. The scalp was shaved and the rat was placed on a stereotaxic apparatus using 45° ear bars to prevent eardrum rupture. The eyes were protected with erythromycin eye ointment. We disinfected the scalp and made an incision to expose the skull surface. A laboratory-made 32-channel electrode array was implanted in the right hemisphere of the dorso-medial striatum (DMS, centered on 1.7 ± 0.5 mm AP, 1.8 ± 0.5 mm ML, -3.5 mm DV from brain surface) and secured with jeweler’s screws and dental cement. The multi-electrode consisted of 8 tetrodes arranged in 2 × 8 arrays and mounted on Omnetics connectors (Neurolinc, Minneapolis, MN, United States). A tetrode was made of 4 twisted FeNiCr wires (diameter, 12.7 μm), which was coated with hard PAC (RO-800, SANDVIK, Stockholm, Sweden). Before being implanted into the brain, the tip of the wires was cut neatly and electroplated with gold (code 5355, Basra, Iraq) to an impedance of 0.4∼0.6 MΩ with a laboratory built multichannel plating device. After the multi-electrode implantation, gentamicin (0.5 mg/100 g) was provided immediately by intraperitoneal injection to prevent post-operative infection. After a week of recovery, rats were gradually reinitiated in the water restriction regime and were then exposed to the recording stage. After the conclusion of neural data recording, rats were euthanized with pentobarbital. A lesion was made by passing current (20 μA, 10 s) through each tetrode to mark the tip of the recording tracks. The rats were then perfused transcardially with 0.1M PBS buffer followed by 4% paraformaldehyde. Transverse sections (60 μm) were cut and the electrode holes were plotted onto a rat brain atlas (Paxinos and Watson, 2007).

### Data Acquisition

Wide-band neurophysiological signals from the implanted electrodes were transmitted to a recording system (OmniPlex, Plexon, Dallas, TX, United States) and processed at gains of 1000. These input signals were then resampled at 40 kHz for acquiring spike activity or 1 kHz for collecting LFP. When the voltage on any of the four channels of a single tetrode exceeded a threshold set by the experimenter, a 1200-μs window of the spike waveform on each of the four channels of the tetrode was recorded to disk and time-stamped with microsecond resolution. During each session, the spike waveforms of each channel represented as a mixture of signals including both neurons and background noise, single units were isolated off-line by clustering spike features recorded on each tetrode with MClust-4.3 (Mclust-4.3, Redish et al.)^1^ and KlustaKwik (Harris). To ensure that spikes sorted to a cluster were well isolated from others, two main quantitative measurements were applied to assess cluster quality. Isolation distance (ID) measured how many sigmas were needed to double the number of points in the cluster, and the L-ratio measured the number of extra-cluster points, weighted by the expected γ2 distribution expected from the Gaussian shaped cluster (Schmitzer-Torbert et al., 2005). Importantly, we set strict criteria to lower the false positive rate, only the neurons with both ID > 15 and L-ratio < 0.05 were accepted for further analysis. At the beginning of the recording stage, the striatum was identified by the presence of slow firing cells and low-frequency LFP (∼10Hz, high-theta oscillations), then each tetrode was moved down forward 35 to 70 μm per day until the estimated depth reached 5.5mm (**Supplementary Figure S1**). We recorded 557 well-isolated DMS neurons from 121 sessions. This sample size was sufficient for the statistical analysis used in this study.

### Behavioral Data Analysis

All data analysis in this study was performed with MATLAB (MathWorks, Natick, MA, United States). At the training stages, mean values and coefficients of variation (CV) of the ATs of each trial within a session were used as the main measurements to assess the subjects’ performance. Each stage was divided into early, intermediate and late phases according to the schedule. Each phase contained three consecutive sessions of data for the corresponding period of the stage. If the CV of the AT distribution was lower than 0.4 (relatively high precision), it was deemed that the rat had already learned to estimate the target time and could be upgraded to next stage. Based on the scalar timing theory, longer ATs are associated with larger variances (Matell and Meck, 2004; Buhusi and Meck, 2005; Merchant et al., 2013; Namboodiri et al., 2014). Hence, the optimal AT for the lowest CV approximates 1.5 s according to the asymmetric set-up of the reward window.

The recording stage started once the well-trained rats had recovered from surgery. Trials showing outlier ATs (<300 ms or >3000 ms) or exiting during the latency period were removed, as it was unlikely that subjects had used the acoustic cue to time the target duration in these trials. Those non-timed trials made up a small percent of the entire database (8.74%, 1773 versus 20294 trials). In order to ensure that stereotyped durations truly timed from the SO and had less dependence on the variant latency, we separated the rewarded trials from the unrewarded trials and explored the relationships among WPI, SO and corresponding WPO. The timing dependent event was tested by linear regression. We extracted randomly 1000 trials from the whole trial pool and tested the divergence (R^2^) of the HT and the AT. These data were sorted by their duration and linear fitted again. We expected the slope (α) of this linear fitting equation to be relatively gentle if the rats timed precisely from a specific event. We assumed that the unrewarded trials were more affected by other behavioral events.

### Neuron Classification

**FIGURE 2:**
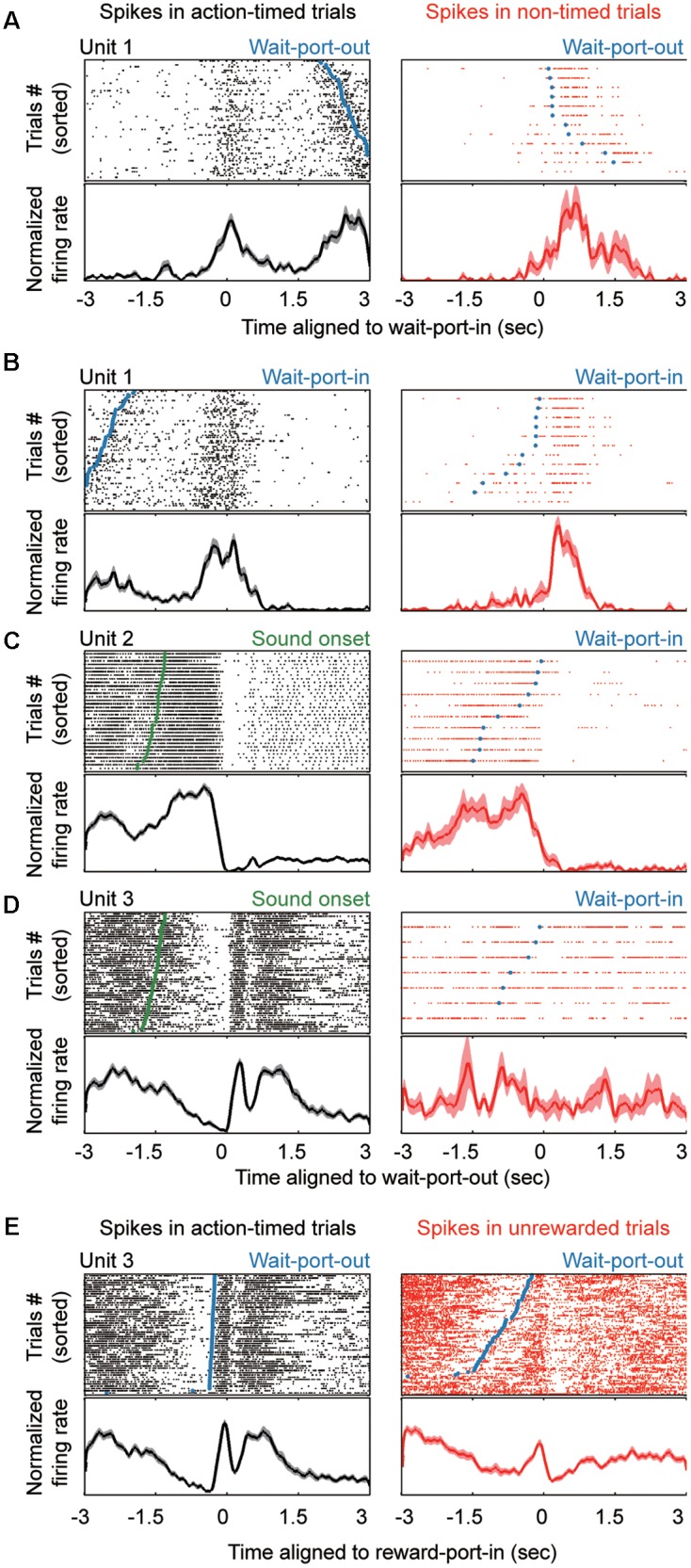
Striatal neurons show variable modulation responses to events in the task. **(A–E)** *Top*, raster plots showing the neural activities aligned to different events during the task. *Bottom*, normalized firing rate for each unit. The left column shows units in action-timed trials, whereas the right column shows units in non-timed trials. Task events are marked in the raster plots, as follows: wait-port-out (**A,E**, blue line or dots), wait-port-in (**B**, blue line or dots), SO (**C,D**, green).

The striatum comprises the projection population of medium-spiny projection neurons (MSNs, GABAergic) and two key types of interneuron subtypes: the fast-spiking interneurons (FSIs, putative parvalbumin containing interneurons) and the tonically active interneurons (TANs, putative cholinergic interneurons) (Kreitzer, 2009; Gerfen and Surmeier, 2011). Because MSNs make up the overwhelming majority of striatal neurons and are characterized by their long spike duration and low firing rate; putative MSNs and putative FSIs could be identified by three parameters: firing rate, peak width (full width of half maximum) and valley width (**Figure 2A**). The calculated waveform parameters for each unit were verified manually. Clusters representing TANs were difficult to distinguish from MSNs and FSIs and therefore, further parameters of firing properties were taken into consideration, including low percentages of spikes occurring in bursts, long post-spike suppression and small percent of long inter-spike intervals (ISI) (Thorn and Graybiel, 2014). A burst event was defined as a series of spikes in which the ISIs were < 10 ms. Post-spike suppression was calculated by constructing an autocorrelation histogram for each unit (range, 0 to 1 s) using 1-ms bins, then the autocorrelogram was convoluted with a 50-point Hamming window. The post-spike suppression was the time when firing expectation first exceeded the average one over the 1 s window (Schmitzer-Torbert and Redish, 2008). The k-means algorithm determined thresholds, and units showing outliers were excluded manually before clustering (**Table 2**).

**Table 2:** Striatal neuron classification.

Putative population	MSNs	FSIs	TANs
Firing rate (Hz)	4.1 ± 0.23	18.9 ± 0.83	3.68 ± 0.42
Peak width (us)	142.4 ± 2.0	93.2 ± 1.2	160.0 ± 12.0
Valley width (us)	561.5 ± 7.9	297.0 ± 4.8	567.1 ± 32.9
% Short ISIs	51.0 ± 1.0	83.1 ± 0.8	26.6 ± 2.8
% Burst	7.3 ± 0.3	17.3 ± 0.8	0.7 ± 0.2
Post-spike suppression (ms)	10.9 ± 0.4	10.9 ± 0.6	65.0 ± 4.7

### Neural Data Analysis

Before analysis of neural activity, the timestamps of each spike from identified single units were aligned to the behavioral events, such as WPI, WPO, SO and reward port entrance (RPI) (**Figure 1A**). Subsequently, peri-event time histograms (PETH) for each putative neuron were first constructed using 20-ms bins, then smoothed (MATLAB, smoothts function) by convolution with a Gaussian kernel (wsize = 10, stdev = 4). Only the rewarded test trials (when the acoustic cue lasted 2.5 s while rats timed a 1.5 s duration and exited from the wait port) in each session were extracted for timing related behavioral analysis.

To assess the effects of different behaviors and responses on neuronal activity, we compared the firing rates between the peri-event window and baseline to determine event-responsive units. A different peri-event window was designed for each behavioral event according to the corresponding neural activity observed in PETH and several time windows were explored. Finally, the firing rate in a window of 200 ms before and after the WPI and WPO across the session was compared to baseline (using the ranksum test). A significant change was judged if the *p*-value was found to be less than 0.01. Baseline firing rate was evaluated using bootstrapping method by sampling, with 1000× replacement, the corresponding window from a session. Testing whether there was a reliable change in firing rate between rewarded and non-rewarded state identified the reward-responsive units. A 400-ms time window following reward-port entrance was considered suitable to attenuate the motor-relevant signals and reflect the reward-relevant signal.

The method to test the modulation during time-estimation period was more complex because the firing profiles had sustained and ramped responses that encoded timing information. In order to remove the trial-by-trial timing variance, timestamp of spikes within a trial were firstly aligned by SO time then linearly scaled by 1.5 s to the WPO time. Those data were then smoothed by convolution with a Gaussian kernel (wsize = 300, stdev = 60) to generate a probabilistic firing density. Spike counts of first and last bins were removed to prevent edge effects. Subsequently, we used 15 consecutive overlapping windows of 100 ms, from the SO to 1.5 s, to test for significant modulations of firing rate with respect to baseline using non-parametric ANOVA (Kruskal–Wallis test). A window of 100 ms was used to obtain the baseline spike counts by replacement 1000× from the available pool of trials (200 ms before SO). The longest streak of consecutive significant modulation and corresponding modulation profile (ramping up, ramping down or sustained modulation) was identified. A unit was classified as an action-timing (AT) unit if it showed long-term (*p* < 0.05 in more than consecutive 0.8 s, exceeding half of the timing period) and monotonic modulation. The sign of modulation determined whether it was excited (ramping up, *n* = 74/557, 13.5%) or inhibited (ramping down, *n* = 39/557, 7%). Each AT unit had been reconfirmed manually by its PETH. Additionally, to confirm the monotonic firing property of AT units (i.e., ramping-up or ramping-down tendency across the timing-estimating period), we multi-compared the difference value of the mean spike counts between two bins (by non-parametric ANOVA analysis), and *z*-scored it by subtracting the mean and dividing by the standard deviation of the series (**Figure 4C**).

Further analysis was applied to examine whether the identified AT units displayed monotonic modulation following the SO or WPI, the latter indicating that the modulation might reflect an elapsed duration or the movement of holding posture. We extracted all excited and inhibited units and separated them according to different latency of trials. Next, 10-ms bins were used to construct the PETHs at the population level, smoothed with a Gaussian kernel (wsize = 50, stdev = 30) allowing comparison of three latency groups (0.5, 1.0, and 1.5 s delay) of spike counts distributions at the temporary resolution of 10 ms. A modulation time was identified if the null hypothesis that three latency group showed similar firing distribution could be rejected at *p*-value of 0.01 under Kruskal–Wallis test.

In order to explore how striatal neurons could encode time information, we quantified the empirical effect of each trial according to correct times in the last 10 trials. PETH aligned to different events was processed and then divided by the empirical level of each trial. Some indexes of observed firing mode were taken into consideration, such as the spread and amplitude of peak firing rate relative to WPO from a smoothed PETH aligned to WPO. The correlations between those indices were tested using linear regression method. The measure of spread was defined as the first timestamp showing neuron modulation until the last one, which was equal to the width between the origin of the ramp and the top. The amplitude was the firing changes from the bottom to the top during time-estimation period (300 ms before WPO was removed to reduce motor interference).

## Results

### Fixed Interval Reproduction Task and Timing Performance

To focus on robust time-guiding action over a sub-second–to–second timescale, we employed an interval reproduction task to measure time performance, which required the subjects to make a timed action that was equivalent in duration to the target one (Allman and Meck, 2012; Xu et al., 2014). Briefly, adult rats were trained to reproduce a fixed interval of 1.5 s following an acoustic cue (a pure tone lasting 1.5 s in instructive trials and 2.5 s in test trials) in sequential blocks of trials (**Figure 1A**). To initiate a trial in our fixed interval reproduction task, rats entered the wait port and held their position. After a pseudorandom delay period, an acoustic cue was presented. The rats were rewarded only if they exited the wait port within the reward window. Thus, the water-restricted rats had to estimate the time since the SO in test trials. We analyzed both the behavioral and electrophysiological responses during test trials.

In the early phase of training stages, the rats remained in the nosepoke for relatively short periods, whether there was a cue (after stage 2) or not. During the training process, their performance improved gradually and approached the target duration with a low CV (**Figure 1B**), providing evidence that the rats had learned the rule for each stage on our training schedule. The cumulative distribution function (CDF) of rats’ AT during the recoding stage showed a tight sigmoidal shape (**Figure 1E**) with a median near to 1.5 s (mean AT = 1.486 s) and a low CV of 0.265. These data indicated that the exiting action was increasingly being timed since the SO and became stereotyped.

We asked whether those stereotyped ATs purely reflected timing from the SO or were independent from the WPI. We observed that the ATs at long-delay trials showed skewed distribution and were significantly shorter than short-delay trials (0.5 s-delay block: 1.73 ± 0.34 s; 1.0 s-delay block: 1.49 ± 0.32 s; 1.5 s-delay block: 1.24 ± 0.35 s; *P* < 0.01; one way ANOVA; **Figure 1F**). A possible explanation of this divergence is that a subset of trials was significantly affected by the latency before the SO. In order to extract the trials that were timed from the SO and were less influenced by the variant latency, we removed the trials showing outlier ATs (<300 ms or > 3000 ms). These non-timed trials also included trials at which the rats exited prior to the SO. Based on the inference that longer delays from the WPI to SO presentation would correspond with shorter ATs, we suggest that the trials with ATs out of the reward window might not be fully timed from the cue or might reflect a bad timing performance. Nevertheless, most trials were within the narrow reward window (650 ms), implying that the AT was well guided by the SO in rewarded trials. Hence, trials including the ones dependent on WPI presented lower variance (AT = 1.49 ± 0.39) and steeper slope (α = 1.27) from trial to trial (see section “Materials and Methods”), and these indices were significantly improved after removal of the unrewarded trials (AT = 1.62 ± 0.17; α = 0.58). An example session is shown in **Figures 1C,D**. Thus, the AT period in rewarded trials reliably conveyed the rats’ estimation about time, and was used to analyze the activity of striatal neurons during the execution of the 1.5-s fixed interval reproduction task as described below.

### Striatal Neurons Exhibited Different Firing Patterns among Putative Subtypes

There is extensive evidence that the associative striatal region (dorso-medial striatum, DMS) receives a high proportion of frontal and limbic inputs (Mailly et al., 2013), and contributes to inter-temporal decision-making (Wang et al., 2013) and goal-directed movements (Shadmehr et al., 2010). In order to explore the mechanisms of striatal neuron encoding of time information, we performed tetrode recordings in the DMS of rats that had shown stable performance in the interval reproduction task. In total, 557 well-isolated and high-quality clusters were deemed to be putative neurons. These striatal neurons exhibited a wide variety of firing patterns aligned with different behavior events (**Figure 2**).

A recurrent concern in time-relevant studies is that the time signals need to be segregated from those that reflect motor factors. Therefore, we examined the neuronal responses during action-timed or non-timed trials in each session. We hypothesized that motor signals primarily correlated with movements (**Figures 2A,B**) and that a robust time estimation signal would be present as a trial-by-trial correlation with the corresponding action. We found that longer neural responses indeed indicated a later exit, and vice versa (**Figures 2C,D**, left columns). On the other hand, such trial-by-trial correlation between action and corresponding firing pattern may not be present on non-timed trials during the same session (**Figures 2C,D**, right columns), while the motor signal is independent of the timing behavior (**Figures 2A,B**, right column). In addition, aligned to the reward-port entry, many neurons (*n* = 180, 32.3%) exhibited a significantly different firing pattern between rewarded trials (action timed trials) and unrewarded trials (premature or omission trials). Such reward responsive neurons showed a transient but obvious high intensity firing (**Figure 2E**), which could not be fully explained as motor behavior. Thus, we identified event responsive units (ERU) by comparing their firing pattern around specific events in relation to baseline (ranksum test, *P* < 0.01; see section “Materials and Methods”). The majority of striatal neurons were modulated around the time of WPI or WPO (WPI: *n* = 207, 37.2%; WPO: *n* = 239, 42.9%; either = 332, 59.6%). We found that 113 single units presented trial-by-trial correlations during the time estimation period, suggesting these units were timing-relevant neuron and subjected to further analysis.

**FIGURE 3:**
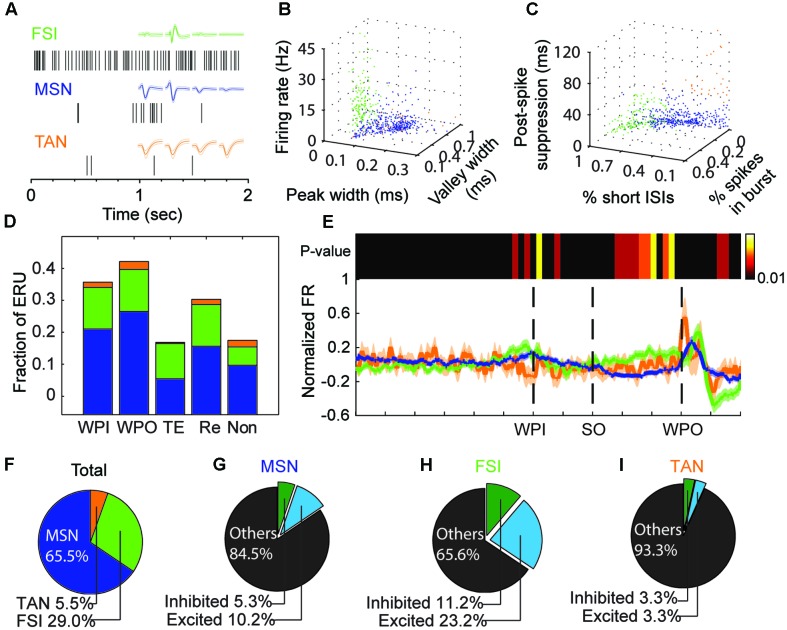
Reponses of different striatal neuron types during the task. **(A)** Representative waveforms and raster data from each striatal subpopulation: FSIs (green), MSNs (blue), and TANs (orange). **(B,C)** Units are clustered into putative MSNs (blue), FSIs (green) or TANs (orange) according to firing rate and waveform properties **(B)**, or bursting properties **(C)**. **(D)** The fraction of event-responsive units (ERU) to different task events, such as wait-port-in (WPI), wait-port-out (WPO), timing event (TE), reward event (Re), or no responsive to those events (Non). Color scheme as in **(A)**. **(E)** *Bottom*, Neural states changes across the task are represented by the mean normalized firing rate (FR, solid line) and standard error (shadow) for each cell type (colors as in **A**). *Top*, heatmap shows the *P*-value of the cell-type-based differences in FRs (bin resolution = 100 ms); a sustained significant difference is clear during the time-estimation period (from SO to WPO). **(F–I)** Pie charts for the all the recorded striatal units (**F**, *n* = 557), MSNs **(G)**, FSIs **(H)**, and TANs **(I)**. The percent of timing-relevant units (excited or inhibited) among cell types are indicated.

The above analysis of striatal neural responses indicated a gross relation between the firing patterns and the corresponding behavior. We asked whether the different types of striatal neurons (*n* = 557 units) showed a preference on responsive events. Cell type classification was performed according to standard criteria (firing rate, waveform and firing property) (**Figures 3A–C**). We labeled 361 units as MSNs (based on the L-ratio, ID and spike counts), of which 78.7% showed firing modulation to at least one event. We labeled 160 units as FSIs, of which 81.9% were accepted as high task-responsive cells. Units classified as TANs (*n* = 30, 5.5% of all units) had 66% task-responsive units. The distribution and fraction of non-responsive units were consistent with previous studies (Kimchi and Laubach, 2009; Thorn and Graybiel, 2014). Next, we compared the distribution of units of a given subtype that were responsive to the same event, in comparison to the total subtype fraction. FSIs showed a significant number of time estimation units (48.7% of FSIs in timing event vs. 29% of FSIs in total; **Figures 3D,F**). This suggests that striatal FSIs exhibit a preference on timing-related modulation of spike activity. Additionally, we found a significant difference in event-specific preference among cell types, especially between MSN and FSI populations (**Figure 3E**), which was confirmed by testing the population neural states across a trial by non-parametric ANOVA (Kruskal–Wallis test, *P* < 0.01) under the window of 100 ms (during the time estimation period). The firing rate of the FSI population increased from the SO until WPO, whereas the firing rate of the MSNs population decreased during corresponding period, which could probably be explained by the GABAergic properties of FSIs.

### Action Timing Was Encoded by the Dynamics of Striatal Subpopulations

As described above, neurons encoding an interval timed from the SO are likely to reflect this information in their firing profile. Consistent with previous studies, two primary profiles were identified during the time estimation period including excited (ramp up) and inhibited (ramp down) response (see section “Materials and Methods”). Although a small group of units (*n* = 42) showed a sustained modulation of spike activity compared to baseline (*p* < 0.01, bootstrap estimate), after manual checking, these units were removed due to their internal variance and poor relevance to the SO. This kind of sustained modulation simply reflects a specific interval rather than the temporal information of elapsed time (by a ramp in firing rate). Of a total of 113 single units showing an obvious monotonic modulation, we found 74 excited AT units vs. 39 inhibited AT units, meaning that 66% of them were excited gradually across the time estimation period. These relative proportions did not differ among cell types (**Figures 3G–I**).

**FIGURE 4:**
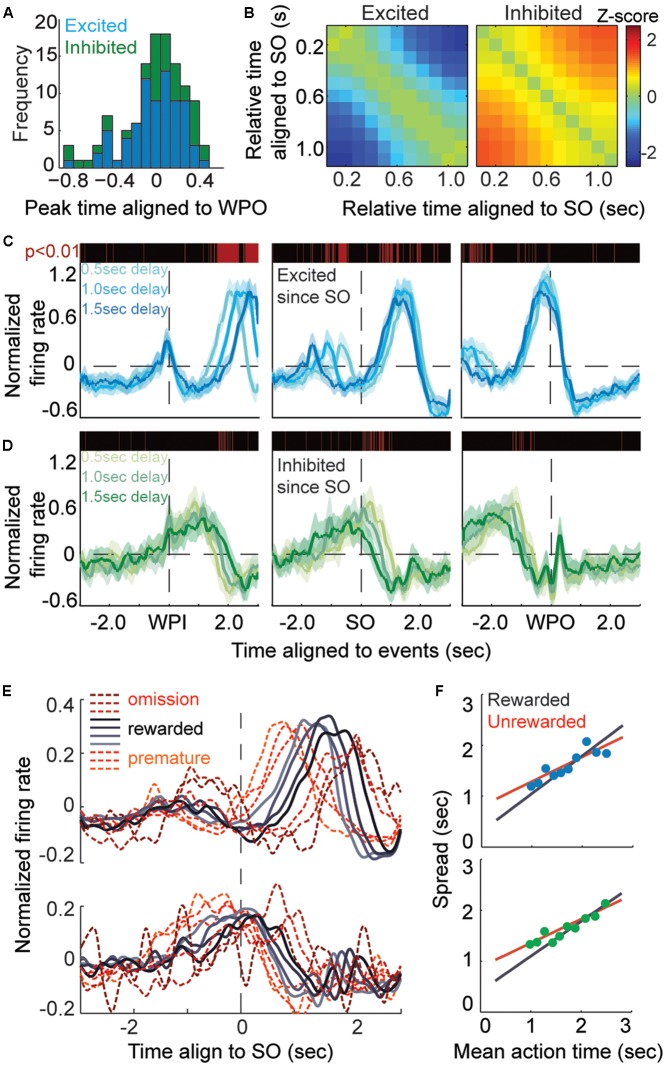
Action-timing neurons display monotonic modulation during the task. **(A)** Frequency distribution of AT units aligned to the WPO, blue bars shows excited units and green bars shows inhibited units. **(B)** Heatmaps of the *Z*-scored changes in firing rate during the time-estimation period (aligned to the SO), which shows the monotonic modulation of TA units, either as excited (left) or inhibited (right) firing rates. **(C,D)** The curves show the averaged event-aligned firing modulation of AT neurons, ramping up (**C**, excited, blue lines) or ramping down (**D**, inhibited, green lines). The *P*-value is tested among separated rewarded trials according to variant latency, a red line means a significant difference (*P* < 0.01) in corresponding 10-ms bins. **(E)** Normalized firing rates aligned to the SO. Trials are divided into 10 groups according to their AT, longer wait times are correlated to longer spreads of ramping activity. **(F)** Plots of the spread of ATs for excited units (top) and inhibited units (bottom). The rewarded groups in AT trials are fitted by the black curve (*R*^2^ = 0.913, α = 0.743), whereas the unrewarded groups in unrewarded trials are fitted by the red curve (*R*^2^ = 0.798, α = 0.484).

A crucial consideration was to ensure that the modulation of firing rate encoded for time rather than ongoing motor patterns, such as holding position. Theoretically, an AT neuron would persistently modulate its firing rate (either exciting it or inhibiting it) from the SO to the termination of target interval, and then the action of exit would follow the moment of last modulation, which is indeed what we found empirically (**Figure 4A**). In other words, those monotonic modulations would be independent of the latency before the cue of ‘start-timing’ (**Figure 4B**). Hence, we separated all the rewarded trials for which the excited and inhibited units had been recorded according to different latency of trials (**Figure 4C**). Only the rewarded trials were used, for two reasons; first, rewarded trials reflected more reliably the AT behavior; and second, the mean AT among groups with variant latency was in proximity to each other (**Figure 1D**), which means that the significant difference reflected only the response time of the acoustic cue. We tested the normalized firing rate within 10 ms among different groups under Kruskal–Wallis test. We found that, when aligned to the WPI, the groups with shorter delay displayed their ramp property at earlier times, but such a divergence was minimized when aligning the groups to the WPO (**Figures 4C,D**).

Next, we quantified the correlation between neural response and action by measuring the spread, equal to the duration of neural modulation during time estimation, and corresponding AT. Both the rewarded and unrewarded trials were aligned to the SO and divided into 10 groups based on the AT (**Figure 4E**). We found that that the spreads of these groups showed a linear correlation (**Figure 4F**) with mean AT of same group (excited population, *R*^2^ = 0.789; inhibited population, *R*^2^ = 0.879). Thus, the dynamics of striatal firing patterns during the time estimation period may directly indicate the AT behavior. In addition, as displayed in **Figure 4F**, both the excited and inhibited subpopulations showed a steeper slope in AT trials than unrewarded trials (excited population, *R*^2^ = 0.913, α = 0.743 in AT trials, *R*^2^ = 0.798, α = 0.484 in unrewarded trials; inhibited population, *R*^2^ = 0.823, α = 0.687 in AT trials, *R*^2^ = 0.952, α = 0.472 in unrewarded trials). The fitted curve slopes gently in unrewarded groups suggesting a migration effect that the premature trials correlated with a longer spread of spike activity modulation while the omission trials correlated with a shorter one. This implies that these trials may be imprecisely timed or not fully dependent on the ‘start-timing’ cue.

### The Amplitude of Monotonic Modulation Encoded the Degree of Timing Assurance

**FIGURE 5:**
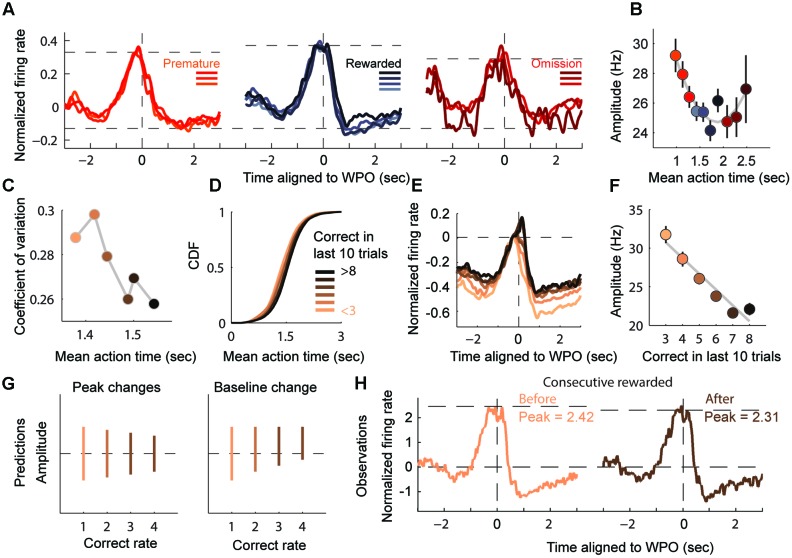
Amplitude of monotonic modulation reflects the degree of timing assurance. **(A)** Normalized firing rates of AT units displaying timing-relevant modulation are segregated into three groups: premature (left), rewarded (middle) and omission (right). **(B)** Plot of the AT amplitudes (mean ± SEM), quantified from the bottom to the peak of the firing ramp (300 ms before the WPO are excluded). **(C)** Plot of the coefficient of variation of the ATs. **(D)** CDF of the ATs for six performance groups (from < 3 to > 8). **(E)** Normalized firing rates of groups in **(D)**, aligned to the WPO. **(F)** Plot of the AT amplitudes (mean ± SEM) for the six groups showing a linear correlation with correct number in recent trials. **(G)** Two predicted patterns for the change in amplitudes. The left panel shows the amplitude changing in absolute terms, while the right panel shows that amplitude changes may also be cause by increasing baseline level. **(H)** Normalized firing rates from consecutive rewarded trials exhibit a declined peak level and no observable shift in baseline.

We have found that the monotonically activation of timing-relevant neurons is scaled to match the current action, and that this dynamics may be ascribed to the internal physical state, not the behavioral act. We next asked whether the neurons could self-calibrate the spread of their firing ramp according to specific length. Recent experimental data have led to the theory of a simple population clock, in which populations of sequentially activated neurons are capable of telling time on seconds to minute scale (Mello et al., 2015). However, such temporal response field cannot fully explain the neural mechanism underlying shorter time scales. As indicated by the diversity of escalating firing rates (**Figure 5A**), the trials in which AT units were closely linked with target duration showed smaller amplitude of firing changes from the SO to 300 ms before exit (**Figure 5B**), compared to the trials with longer mean ATs. We decided to exclude the 300-ms period before the WPO to reduce the potential interference caused by robust movement or smooth process.

We predicted that the amplitude of monotonic modulation encoded the animals’ internal assurance of inter-temporal decision. To test this prediction, we assessed the assurance of each trial according to its previous rewarded experience. It is reasonable to expect that the degree of assurance would compare with the experience of correction, which equals to the rewarded times of preceding trials. As expected, more experienced trials showed more stereotyped (lower CV) and more accurate (close to 1.5 s) timing performance (**Figure 5C**). Furthermore, as the number of correct trials increased, the sigmoidal shape of the CDF of ATs increased more steeply, indicating that the AT gradient was being timed assuredly at target duration (**Figure 5D**). When presented this way, the monotonic modulation of the time estimation period appeared to evoke a smaller amplitude when the correct number was relatively common, as illustrated by linear regression analyses with correlation coefficient *R*^2^ = 0.93 (**Figures 5E,F**). There was a robust jump in activity around the WPO event, especially during highly experienced trials, but the stereotyped short interval between WPO and reward port entry in these trials suggested that these extra firing changes were likely caused by motor components.

Since the target duration and reward magnitude were constant in our task, the variable amplitudes of the time-estimation periods could imply two different scenarios (Schultz et al., 1997; Fiorillo et al., 2003; Hamid et al., 2015), one in which there is a decline from a fixed baseline to a variable level (encodes the assurance to upcoming reward) or a second one with an escalating baseline to a fixed level (encodes state values of motivation) (**Figure 5G**). In order to elucidate this situation, we compared the amplitude in consecutive rewarded trials. Our observation was consistent with the first prediction that the amplitude of modulation decreased with repeated rewards while the baseline remained stable (**Figure 5H**). The ramping down subtype of timing-relevant neurons showed similar but relatively weaker correlation compared with the ramping up ones (data not shown). Overall, we provided further evidence that timing-relevant striatal neurons calibrated the spread of their firing pattern by rewarded experience.

## Discussion

Timing is a fundamental cognitive function to predict and drive future actions. For this reason, it is indisputably generated from multiple brain regions engaging the cognitive or sensorimotor functions. Many electrophysiological studies support the view that sustained neural activity across the brain correlates with elapsed duration. Some classic investigations in patients with neurodegenerative disorders in basal ganglia provide corroborating evidence for the centrality of the striatum in timing voluntary actions. These studies reported that patients with Parkinson’s disease (Koch et al., 2004; Harrington et al., 2011; Jones and Jahanshahi, 2011; Parker et al., 2013), Wilson’s disease (Xu et al., 2010; Jiang et al., 2011), autism (D’Cruz et al., 2009) and ADHD (Valera et al., 2010) often exhibit timing dysfunctions. We recorded from the striatum because, structurally, it is the input layer of basal ganglia that receives glutamatergic afferents from the entire cortex and thalamus, as well as dopaminergic afferents from midbrain; this convergence leads to multiplex temporal information from widespread areas that comprise the internal clock (Kreitzer, 2009; Adler et al., 2012; Reig and Silberberg, 2014). Functionally, the striatum is associated with reinforcement learning and procedural motion, which require the prediction of future events (such as rewards and punishments) and programing the consequences of actions through time; therefore, the striatum needs to process temporal information (Schultz et al., 1997; Lau and Glimcher, 2008; Rueda-orozco and Robbe, 2015). Indeed, most motor tasks require different actions timed in order or to respond to an external stimulus. Such temporal relations are gradually learned through a balance between conflicting psychological factors, such as motivation and reward prediction, the former motivates the animal to hold and cost, while the latter derives pleasure from immediate consumption (Namboodiri et al., 2014; Hamid et al., 2015). These processes are first learned then become automatic on sub-second–to–second range. Our study provides experimental data to confirm the role of striatum in representing AT information.

In our timing behavior task, the brain process of observed stereotyped timing behavior could be decomposed into four consecutive phases: sensing the acoustic cue, estimating a stereotyped interval, producing the action of exit, and then calibrating the internal timing rate regarding the outcome. To address whether the neural activity reflected time or motor patterns in such temporal resolution, we trained the rats to hold their posture in nosepoke to reduce most alternative behaviors, such as grooming or rotating. By inserting a pseudorandom delay period between the salient motor behavior and the ‘start-timing’ cue, we were able to identify the timing signal based on the fact that monotonic firing patterns were significantly modulated during the time-estimation period, rather than the entire posture-holding period. Consistent with observations from other areas of brain, such timing-relevant modulation dynamically rescaled with anticipated duration and instructed the action of exit (Maimon and Assad, 2006; Xu et al., 2014; Mello et al., 2015; Namboodiri et al., 2015). Interestingly, it is worth noting that the divergent slopes of the fitted curves between rewarded and unrewarded trials offer a psychological explanation for the imprecisely timed trials, and the longer spread of modulation suggested that premature actions might be internal timed.

Furthermore, the firing rates of putative timing units in the striatum have been reported to escalate to a threshold under some conditions (Matell et al., 2003; Bartolo et al., 2014; Gouvêa et al., 2015; Mello et al., 2015), but these dynamics might merely reflect the elapsed time rather than the action-guiding delay. Due to the unique design of our task, in which different ATs within reward window led to constant reward magnitude, the reward value was only influenced by the outcome of recent actions (Fiorillo et al., 2003). Consistent with this expectation, our data indicated that the gradient firing patterns during time-estimation periods indeed conveyed empirical information to calibrate the speed of the internal clock. Local circuits within striatum may play a primary role in shaping such dynamics. It is important to note that our study has shown evidence of calibration over sub-seconds to seconds timing, and did not claim that such mechanism was suitable for a longer timescale. In fact, recent studies suggest that durations of seconds to minutes are represented as a population of sequentially activated neurons with variable temporal response fields (Mello et al., 2015).

In our study, putative FSI striatal neurons showed a great functional homogeneity on timing behavior. Compared to interneurons, MSNs showed a more generalized response, especially tuned to motor actions. Such cell-type difference may be crucial for timing synchronization within the striatal local circuits. Striatal FSIs receive excitatory synapses from the cortex and the thalamus and are much easier to be activated than MSNs. FSIs can also be found in cortex and hippocampus, and some studies demonstrate their significant role in attention (Kim et al., 2016) and procedural learning (Mallet, 2006). Considering that the putative FSIs were identified by their waveforms and electrophysiological properties, we cannot claim that timing-relevant modulation in our task is exclusively or even predominately encoded by the FSI population. Further pharmacological and optogenetic experiments are needed to confirm these results. Nevertheless, to our knowledge, this is the first study to correlate a cell type with a specific timing process. The finding that FSI, but not MSN, population represents the timing preference modulation in striatum suggests a new explanation to the clinical phenomenon that patients with Parkinson’s disease and certain psychiatric disorders usually perform timing tasks normally on the sub-second scale (Spencer and Ivry, 2005; Wearden et al., 2008). Understanding and manipulating neural functions involved in temporal dimensions is significant both in scientific and clinical fields. Further studies are needed to elucidate the fundamental mechanisms of the timing system at the cell type level; as such information could be used to improve current neuromodulation technology such as deep brain stimulus (Hamani and Temel, 2012) and other non-invasive brain stimulation (Benninger and Hallett, 2015).

## Author Contributions

All the three authors participated sufficiently in the work to take public responsibility for all or part of the content: X-PW worked for the main conception and design and BS worked for acquisition of data and analysis of data. Z-RW worked for the scientific direction and design. All contributed to drafting this article and revising it.

## Conflict of Interest Statement

The authors declare that the research was conducted in the absence of any commercial or financial relationships that could be construed as a potential conflict of interest. The reviewer JS and handling Editor declared their shared affiliation.

## Footnotes

1 http://redishlab.neuroscience.umn.edu/MClust/MClust.html

## Supplementary Material

FIGURE S1 Recording coordinates for each rat. **(A–D)** Left column of each panel shows the micro-drive electrode recording process and the putative corresponding coordinates, the location of the electrodes were confirmed post-mortem in the 4 rats, with the electrode tips within the proper depth. LFP spectral density acquired during the first recording session in the dorsal striatum reveals high power theta wave (10 Hz).
