# Supplementary figures and images for: The Fast Spiking Subpopulation of Striatal Neurons Coding for Temporal Cognition of Movements
Source: Front Cell Neurosci. 2017 Dec 15;11:406. doi: 10.3389/fncel.2017.00406 (PMC5736568; doi:10.3389/fncel.2017.00406)

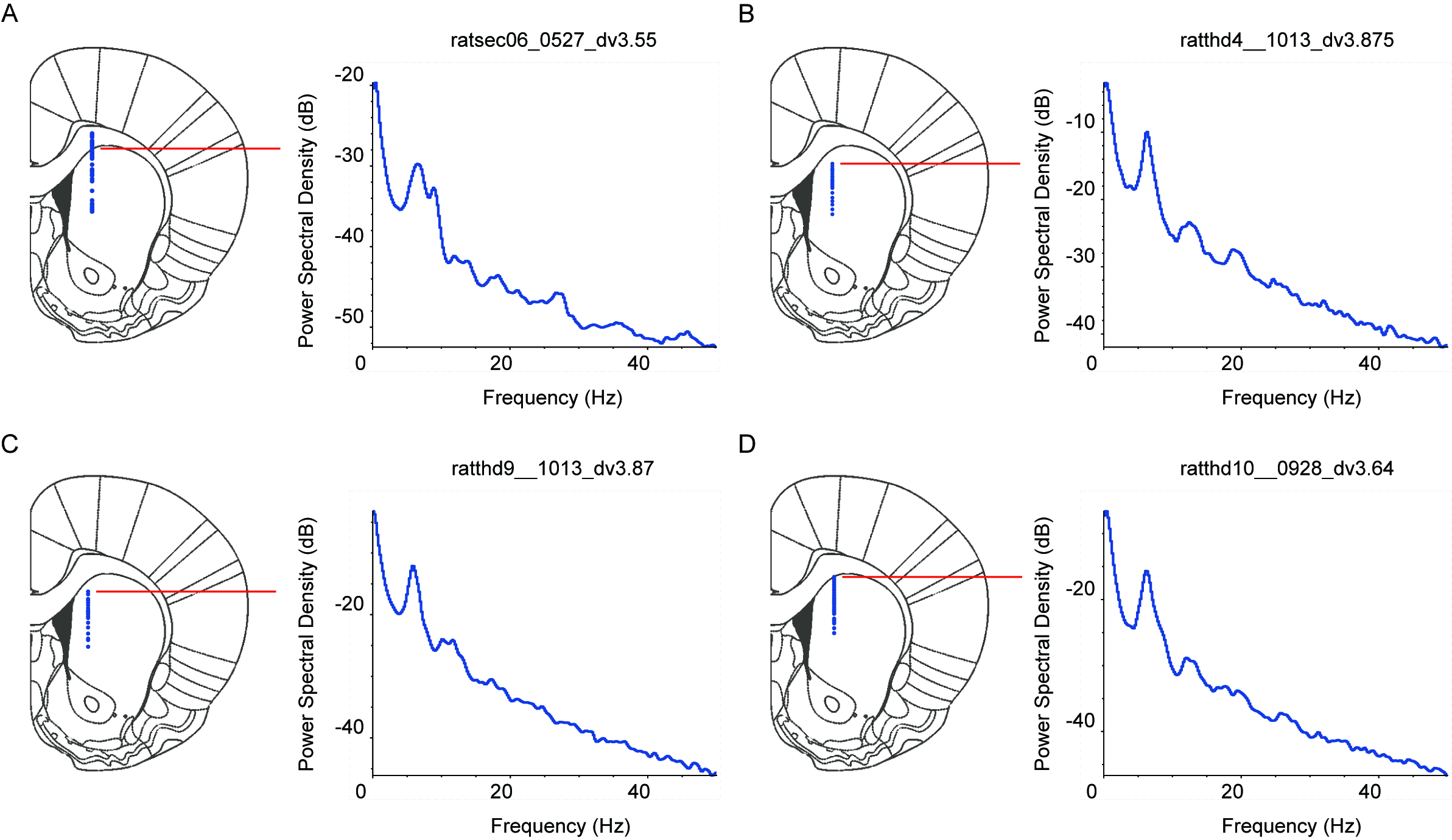

Supplement: FIGURE S1 — Recording coordinates for each rat. (A–D) Left column of each panel shows the micro-drive electrode recording process and the putative corresponding coordinates, the location of the electrodes were confirmed post-mortem in the 4 rats, with the electrode tips within the proper depth. LFP spectral density acquired during the first recording session in the dorsal striatum reveals high power theta wave (10 Hz). [file Image_1.TIF]
